# A PCA–EEMD–CNN–Attention–GRU–Encoder–Decoder Accurate Prediction Model for Key Parameters of Seawater Quality in Zhanjiang Bay

**DOI:** 10.3390/ma15155200

**Published:** 2022-07-27

**Authors:** Zaimi Xie, Zhenhua Li, Chunmei Mo, Ji Wang

**Affiliations:** 1 School of Mathematics and Computer Science, Guangdong Ocean University, Zhanjiang 524088, China; 2112009010@stu.gdou.edu.cn; 2 Guangdong Engineering Technology Research Center of Intelligent Ocean Sensor Network and Equipment, Zhanjiang 524088, China; zhenhuali@gdou.edu.cn (Z.L.); 2112010019@stu.gdou.edu.cn (C.M.); 3 College of Electronic and Information Engineering, Guangdong Ocean University, Zhanjiang 524088, China

**Keywords:** seawater, water quality prediction, attention, principal component analysis algorithm, ensemble empirical modal decomposition algorithm, sea area near Zhanjiang Bay

## Abstract

In order to effectively solve the problem of low accuracy of seawater water quality prediction, an optimized water quality parameter prediction model is constructed in this paper. The model first screened the key factors of water quality data with the principal component analysis (PCA) algorithm, then realized the de-noising of the key factors of water quality data with an ensemble empirical mode decomposition (EEMD) algorithm, and the data were input into the two-dimensional convolutional neural network (2D-CNN) module to extract features, which were used for training and learning by attention, gated recurrent unit, and an encoder–decoder (attention–GRU–encoder–decoder, attention–GED) integrated module. The trained prediction model was used to predict the content of key parameters of water quality. In this paper, the water quality data of six typical online monitoring stations from 2017 to 2021 were used to verify the proposed model. The experimental results show that, based on short-term series prediction, the root mean square error (RMSE), mean absolute percentage error (MAPE), and decision coefficient (R^2^) were 0.246, 0.307, and 97.80%, respectively. Based on the long-term series prediction, RMSE, MAPE, and R^2^ were 0.878, 0.594, and 92.23%, respectively, which were all better than the prediction model based on an enhanced clustering algorithm and adam with a radial basis function neural network (ECA–Adam–RBFNN), a prediction model based on a softplus extreme learning machine method with partial least squares and particle swarm optimization (PSO–SELM–PLS), and a wavelet transform-depth Bi–S–SRU (Bi-directional Stacked Simple Recurrent Unit) prediction model. The PCA–EEMD–CNN–attention–GED prediction model not only has high prediction accuracy but can also provide a decision-making basis for the water quality control and management of aquaculture in the waters around Zhanjiang Bay.

## 1. Introduction

With the rapid development of human society and the increasingly serious situation of marine pollution, mariculture is in demand for the world’s aquatic products [1]. Taking mariculture in Zhanjiang Bay as an example, Cheng Haiou et al. [2] conducted an all-round in-depth study on water eutrophication in the bay and found that the rapid increase in nitrogen and phosphorus contents indirectly led to abnormal and severe eutrophication of seawater, which is extremely harmful to mariculture. With the industrialization and urbanization of human beings, the pollution of water in land estuaries is becoming more and more serious, the minerals are increasing, and the eutrophication is intensifying, so it is important to grasp the change pattern of water quality timely and accurately to prevent the deterioration of water quality and the disease outbreak of farmed economic animals. The quality of water used in aquaculture will directly affect the growth and harvest time of fish [3,4]. Seawater quality is susceptible to the metabolism of aquatic organisms as well as anthropogenic activities and is characterized by nonlinearity, instability, and spatial and temporal distribution [5].

The quality of aquaculture water is directly related to the safety of aquaculture, and it is of great value to study the trends of aquaculture water quality parameters to achieve an accurate prediction of water quality parameters and to solve fish disease outbreaks caused by water quality deterioration. Therefore, the prerequisite for constructing an accurate water quality prediction model is to have good water quality data, and further preprocessing of water quality sample data is required before training. A. SslX et al. [6] and X. Sun et al. [7] used a PCA algorithm to correlate seawater quality and reduce the dimensionality of water quality data, respectively. P. Pandey et al. [8] and N. Raj et al. [9] used an EEMD algorithm to decompose seawater water quality data, with noise reduction processing and superposition of each signal after decomposition, so that the model could extract complex and noisy water quality data features to improve the computational efficiency of the model. Y. Zhang et al. [10] used the Kernal Principal Component Analysis (KPCA) method to reconstruct water quality information to improve the training efficiency and prediction accuracy of the model in order to reduce the noise of the original water quality data and retain the original water quality information. The comprehensive research above shows that the EEMD algorithm can decompose the original data and denoise and fuse the component sequences, and the PCA algorithm can exclude the factors that have less influence on water quality variables and reduce the dimensionality of the water quality data.

At present, some scholars at home and abroad have conducted research on seawater quality prediction methods. D. Li et al. [11] proposed the ECA–Adam–RBFNN prediction model to predict the dissolved oxygen content of seawater, and the experimental results showed that the three metrics MAPE, MAE, and RMSE were 0.0052, 0.0385, and 0.0472, respectively. S. Cao et al. [12] constructed the PSO–SELM–PLS prediction model, and the experimental results showed that the three metrics RMSE, MAPE, and MAE were 0.270, 0.0371, and 0.2284, respectively. L. Wang et al. used the PSO–SELM–PLS prediction model, and the experimental results showed that the three metrics RMSE, MAPE, and MAE were 0.270, 0.0371, and 0.2284, respectively. L. Wang et al. [13] and J. Liu et al. [14] proposed M-DJINN and wavelet transform-depth Bi–S-SRU prediction models, respectively, and the proposed models achieved 96% prediction accuracy. A. Bilali et al. [15] developed stochastic gradient descent for linear regression (SGD), an artificial neural network (ANN), k-nearest neighbors (k-NN), and support vector machine (SVM) prediction models for chloride concentration and the sodium adsorption ratio using physical parameters as inputs to the models, and SGD and ANN outperformed the other methods in terms of R^2^ and RMSE. J. Jiang et al. [16] constructed a support vector machine (SVR), deep neural network (DNN), and RandomForest, ElasticNet, and XGBoost prediction models to determine the abundance of *Vibrio vulnificus* in estuarine and mariculture areas, with input parameters of temperature, salinity, dissolved oxygen, pH, total nitrogen, and total phosphorus, and used SHapley Additive exPlanations (SHAP) to analyze salinity and temperature as the main influential factors of *Vibrio* abundance, proving that DNN and RandomForest outperformed the other methods in two evaluation indexes, RMSE and MAE. H. Kim et al. [17] used a regression tree, support vector regression (SVR), bagging, RandomForest, gradient-boosting machine (GBM), and extreme gradient boosting (XGBoost) for the prediction of chlorophyll a. Some physical, chemical, and biological factors affecting chlorophyll a were used as inputs to the model, and XGBoost outperformed the other models in terms of MAE, RMSE, and R^2^. B. Er et al. [18] established an artificial neural network for the prediction of total coliform values with input parameters such as pH, dissolved oxygen, temperature, conductivity, salinity, biological oxygen demand (BOD), total suspended solids, ammonia nitrogen, chlorophyll a, and heavy metals, and the neural network model was able to best predict marine pollution. The above studies solved the problems of local convergence and parameter optimization of water quality prediction models, and the accuracy of short-term time series prediction of water quality was significantly improved. However, there are still shortcomings in the improvement of the accuracy of medium and long-term time series prediction of water quality.

With the development and improvement of machine learning methods, deep learning methods are widely used in various fields; especially in the field of water quality prediction, the method can extract medium-term water quality time and spatial series features. The LSTM network structure with a forgetting gate and an update gate can well excavate the time series characteristics of water quality. C. Xiao et al. [19] used the advantages of LSTM, combined with a strong predictive ability, and reported that it was not easy to overfit the AdaBoost method. Z. Hu et al. [20] first used linear interpolation and other pre-processing methods to denoise the water quality data, based on which the LSTM prediction model was built to predict water temperature and pH, solving the shortcomings of RNN and other traditional methods with low accuracy and high time requirements. The prediction accuracies of pH and water temperature were 98.56% and 98.97%, respectively, for short-term series and 95.76% and 96.88% for long-term series. To further improve the accuracy of water quality prediction, the above study needs further exploration in terms of the extraction of water quality spatial features.

Therefore, the extraction of water quality spatial sequence parameter features is considered, and many researchers have used the advantages of convolutional neural networks to extract water quality spatial features. C. Xiao et al. [21] proposed a convolutional long- and short-term memory model for sea surface temperature prediction, and the prediction accuracy and convergence performance were significantly improved compared with SVR and two-layer LSTM models. M. Han et al. [22] constructed a convolutional neural network model to evaluate sea surface temperature and salinity for 12 months, in which the prediction accuracy of four months, January, March, July, and August, was 96.8%, 97.1%, 94.9%, and 96.7%, respectively, and the research method was able to improve the prediction accuracy of sea surface temperature for each month. The above study solved the problem of the spatial feature extraction of water quality parameters, and the accuracy of water quality prediction was significantly improved. However, in the process of water quality feature extraction, the selection of important features of water quality and the change of the hidden state of the prediction model at each moment of dynamic learning were ignored.

To further consider the selection of important features of water quality and the dynamic learning of the change of the hidden state of the model at each moment, researchers have added attention mechanisms to models, which are divided into a temporal attention mechanism and a spatial attention mechanism. J. Xie et al. [23] used the temporal attention mechanism combined with the GED network to construct the Attention–GED prediction model for sea surface temperature prediction; the temporal attention mechanism can determine the influence of hidden states in the GED network in each time window and improve the prediction accuracy of the model. Y. Liu et al. [24] proposed a prediction model integrating the temporal attention mechanism and spatial attention mechanism with the LSTM network, which can well capture the water quality characteristics of long- and short-term sequences, but there are two drawbacks: on the one hand, the spatial attention mechanism and deep learning methods combined with the model have not yet been used in the field of seawater quality prediction; on the other hand, with the further growth of the prediction sequence, the existing methods for seawater quality with series that include different periods and the spatial series of key parameter feature extraction and prediction accuracy improvement have shortcomings.

In summary, this paper uses the PCA algorithm to reduce the dimensionality of water quality features, the EEMD algorithm to reduce water quality noise, CNN to extract water quality spatial features, attention for learning water quality external features and hidden state information, a self-encoder to achieve an automatic selection of water quality features, and GRU with a reset gate and an update gate combined with a codec algorithm, which has the advantage of efficient and accurate access to water quality feature information, to construct a CNN–Attention–GED model to predict the key parameters of the water quality spatial–temporal sequence.

## 2. PCA–EEMD–CNN–Attention–GED Prediction Model

**Figure 1:**
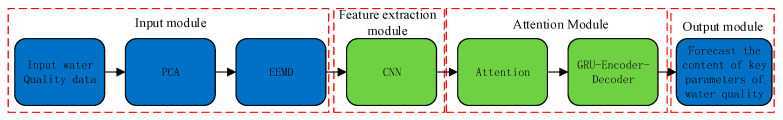
Flow chart of the prediction model.

In this paper, based on data pre-processing algorithms, CNN, Attention, and GED networks, we construct a seawater quality spatial–temporal sequence key parameter prediction model, which contains four modules, namely, network input module, feature extraction module, attention module, and network output module, as shown in Figure 1. The network input module is mainly for the original water quality data noise reduction and dimensionality reduction to improve the network for learning water quality data features and computational efficiency. The feature extraction module mainly uses a convolutional neural network to extract water quality local features; the attention module uses a spatial–temporal attention mechanism to make the GED network dynamic learn the water quality feature information and hidden state of each moment, and the network output module mainly uses the network to complete the learning of the water quality features and further predict the content of the key water quality parameters. 

### 2.1. Network Input Module

**Figure 2:**
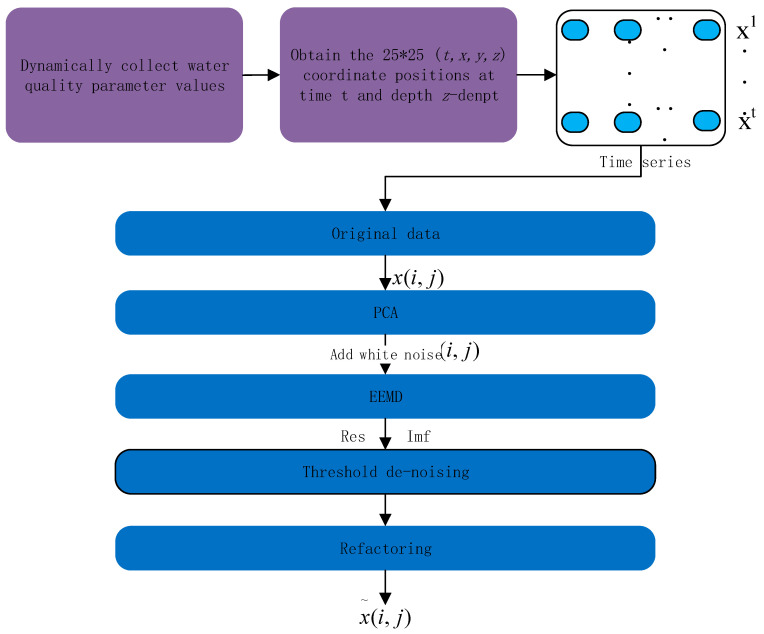
Network input module.

The network input water quality data are characterized by volatility, seasonal periodicity, trend, low correlation, nonlinearity, and dimensional inconsistency; therefore, the network input module is used to pre-process the seawater quality data, and the structure is shown in Figure 2. The original data form a four-dimensional vector, that is, the content of each parameter at time t and depth *z-dept* at 25 × 25 (*t, x, y, z-dept*) coordinate positions, which is input to the PCA algorithm for dimensionality reduction. The processed data will be calculated to obtain five major principal components that are input to the EEMD algorithm for processing; the algorithm is an adaptive nonlinear time-varying signal decomposition. White noise will be added to the original multiple times signal, which ensures the continuity of the signal at different scales and prevents the phenomenon of aliasing. The water quality data were decomposed into 28 eigenfunctions (IMF) by the EEMD algorithm, using the threshold method for each feature signal noise reduction process, reconstructing the noise reduction signal, to obtain the noise reduction water quality data (i,j)x˜.

To the input raw water quality data *x*(*i*, *j*), add the white noise signal *n_k_*(*i*, *j*), as shown in Equation (1).

*x_k_*(*i, j*) = [*x*(*i*, *j*) + *n_k_*(*i*, *j*)] *k* =1, 2, …, *M* (1)

The white noise signal *x*(*i*, *j*) is decomposed into a number of IMFs and a RES by *t* real trials, *c_t,k_*(*i*, *j*) is the *k*th IMF of *x*(*i*, *j*) in *M* trials, *r_L,k_*(*i*, *j*) is the residual, and *L* is the maximum number of IMFs in each trial, as shown in Equation (2).

x_k_(i,j) = ∑ 𝑐𝑡,𝑘 (𝑖,𝑗)+ 𝑟𝐿,𝑘 (𝑖,𝑗) 𝐿 𝑡 = 1 k = 1, 2, ..., M (2)

The mean values of IMF and residual Res for *M* trials were calculated as in Equations (3) and (4).

(3) $$\overset{-}{c_{t}}(i,j)=\sum_{k=1}^{M}c_{t,k}(i,j)/M$$

(4) $$\overset{-}{r_{L}}(i,j)=\sum_{k=1}^{M}r_{L,k}(i,j)/M$$

For signal noise reduction, $\tilde{c_{k}}$ is the threshold value of the kth IMF corresponding to the threshold value *Th_k_*; all IMFs were reconstructed to obtain the noise-reduced data, i.e., (i,j)x˜, as shown in Equations (5)–(8).

(5) $$\begin{array}{l} E_{k}={\left(median(|\overset{-}{c_{t}}|)/0.6745\right)}^{2}\\ k=1,2,\ldots ,L \end{array}$$

(6) $$\tilde{c_{k}}=\{\begin{array}{c} sgn\left(\bar{c_{k}}\left(i,j\right)\right)\left(\left|\bar{c_{k}}\left(i,j\right)-Th_{k}\right|\right)\;\\ \left|\bar{c_{k}}\left(i,j\right)\right|\geq Th_{k}\\ 0\;\left|\bar{c_{k}}\left(i,j\right)\right|<Th_{k} \end{array}\;$$

(7) $$Th_{k}=E_{k}\sqrt{2\ln(p)}$$

(8) $$\tilde{x}\left(i,j\right)=\sum_{k=L}^{L1}\tilde{c_{k}}\left(i,j\right)+\sum_{k=L1+1}^{L}\bar{c_{t}}\left(i,j\right)+\bar{r_{L}}\left(i,j\right)$$

### 2.2. Feature Extraction Module

**Figure 3:**
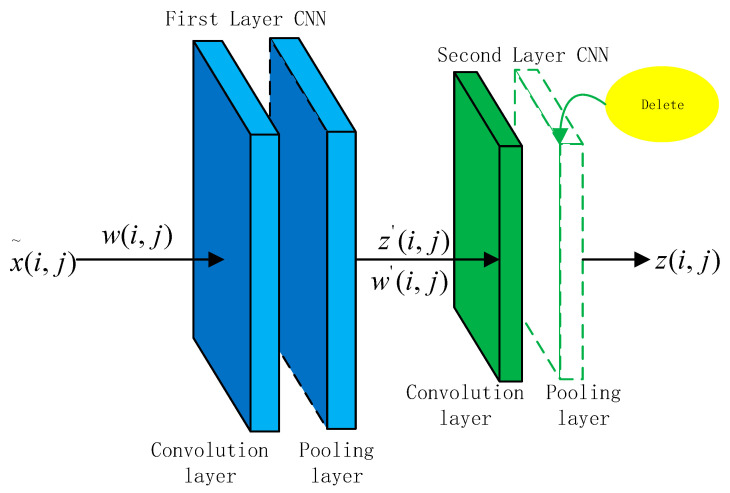
Feature extraction module.

The feature extraction module mainly extracts the local features of water quality data, so that the convolutional layer in the convolutional neural network can learn water quality local feature information; the structure is shown in Figure 3. The processed original data *x*(*i*, *j*) are used as the input of the two-dimensional convolutional neural network [25] (2D-CNN). The convolutional network layer is set to 2 layers: the first layer of the network structure includes the convolutional layer and pooling layer, and the second layer does not contain the pooling layer, but the convolutional layer is retained to improve the network computational efficiency. The last layer is the fully connected layer. The two-dimensional convolutional neural network has four dimensions, the structure is shown in Figure 3. If the input of the 2-dimensional convolutional layer is (i,j)x˜, then the signal characteristics are obtained through (i,j) x˜ with *a × b* of the convolution kernel *w*(*i*, *j*) to obtain *z*(*i*, *j*), as shown in Equation (9).

(9) $$z(i,\;j)=\tilde{x}(i,\;j)\times w(i,j)=\sum_{s=0}^{a-1}\sum_{k=0}^{b-1}x_{t}\left(s,k\right)w\left(i-s,j-k\right)$$

### 2.3. Attention–GED Attention Module

**Figure 4:**
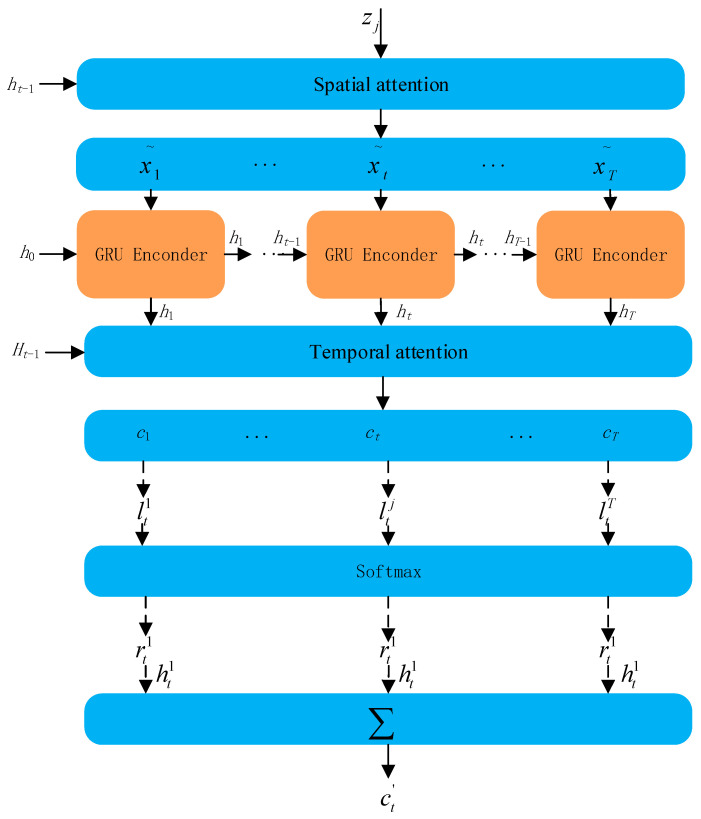
Attention module.

Water quality parameters interact with each other, adding an attention mechanism that can be used to extract water quality spatial–temporal sequence features as input for GED network learning; the structure is shown in Figure 4. The attention mechanism is divided into the temporal attention mechanism and the spatial attention mechanism. The two-dimensional convolutional neural network output of the three-dimensional vector data as the input of the spatial attention mechanism module can dynamically learn the spatial features extracted by the convolutional neural network *z*(*i*, *j*), generating the corresponding weights (*h_j_*) vector. These data are input into the GRU network for encoding, and the encoded data are used as the input of the temporal attention mechanism module, dynamically learning the change in the hidden state at each step, generating the corresponding weights *c_t−_*_1_.

*z_j_*$\in R^{n\times T}$ is the weighted attribute, *h_j_* $\in R^{m\times T}$ is the hidden state, *v_d_, b_d_* ∈ R^m+n^, *w_d_* $\in R^{\left(m+n\right)\times 2q}$, and *u_d_* $\in R^{\left(m+n\right)\times \left(m+n\right)}$ are parameters for model learning, *q* is the number of hidden states, and $H_{t-1}^{0}\in R^{q}$ and $c_{t-1}^{0}\in R^{q}$ are the respective hidden states and cell states of the GRU encoder. $h_{t}^{\prime }$ = [*z_t_*;*h_t_*] $\in R^{\left(m+n\right)\times T}$ represents the joint spatial vector relations, [*H_t_*_−1_;*c_t_*_−1_] represents the temporal vector relations, and *c_t_* denotes the spatial–temporal relations within a time window, as shown in formula (10)–(13).

(10) $$l_{t}^{j}=v_{d}{}^{T}\sigma_{c}(w_{d}[H_{t-1};c_{t-1}]+u_{d}[z_{t};h_{j}]+b_{d})j\in [1,T]$$

(11) $$r_{t}^{j}=\sigma_{z}(l_{t}^{j})j=1,2,\ldots ,T$$

(12) $$\tilde{h_{t}}=(r_{t}^{1}h_{t}^{1},r_{t}^{2}h_{2}^{1},\ldots ,r_{t}^{T}h_{T}^{1})$$

(13) $$c_{t}^{\prime }=\sum_{j=1}^{T}r_{t}^{j}\tilde{h_{t}}$$

Next, the temporal attention mechanism and the spatial attention mechanism mentioned in Section 2.3 are elaborated with their intrinsic mechanisms in Section 2.3.1 and Section 2.3.2.

#### 2.3.1. Temporal Attention

Each cell of the model stores temporal information through a cellular mechanism and controls the increase or decrease of temporal information through a gating cell so that continuous dependence can be maintained. The temporal attention mechanism is used to learn the hidden cell states in the time window. The attention weights of the hidden states at moment *t* are calculated as follows.

(14) $$\delta_{t}^{k}=v_{d}^{T}\sigma_{c}(W_{d}[H_{t-1},c_{t-1}]+U_{d}x^{k}+b_{d})$$

(15) $$\rho_{t}^{k}=\sigma_{z}(\delta_{t}^{k})$$

(16) $$c_{t}=\sum \rho_{t}^{k}h_{k}$$

**Figure 5:**
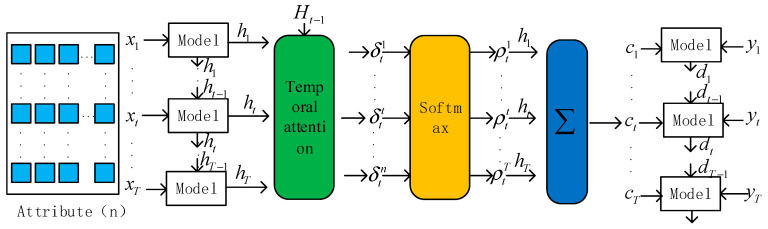
Temporal attention.

*v_d_*, *b_d_*, *W_d_*, and *U_d_* are the variables to be learned.$\;\sigma_{z}$, *d_t_*_−1_, and *c_t_*_−1_ are used as the respective activation function (softmax function), hidden state, and cell state of the previously hidden cell of the model in temporal attention, and *c_t_* is the cell state computed in each time window of the model cell that needs to be obtained, the temporal attention structure is shown in Figure 5.

#### 2.3.2. Spatial Attention

The spatial attention mechanism focuses on learning the spatial correlation between external attributes of water quality. The sum of the attention weights is 1, not ignoring the influence of any external attributes, and does not allow any low correlation attributes to interfere with the water quality prediction results. If given *k* external attributes *x^k^* = {$x_{1}^{k}$,$\;x_{2}^{k}$,…, $x_{T}^{k}$}, the specific formulas are as follows:

(17) $$\eta_{t}^{k}=v_{f}^{T}\sigma_{c}(W_{f}[h_{t-1},c_{t-1}]+U_{f}x^{k}+b_{f})$$

(18) $$\partial_{t}^{k}=\sigma_{z}(\eta_{t}^{k})$$

(19) $$\tilde{x_{t}}={\left(\partial_{t}^{1}x_{t}^{1},\partial_{t}^{2}x_{t}^{2},\ldots ,\partial_{t}^{n}x_{t}^{n}\right)}^{T}$$

**Figure 6:**
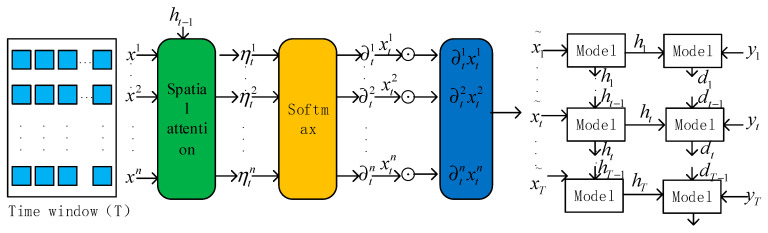
Spatial attention.

*v_f_*, *b_f_*, *W_f_*, and *U_f_* are the variables to be learned.$\;\sigma_{z}$, *h_t_*_−1_ and *c_t−_*_1_ are used as the respective activation function (softmax function), hidden state, and cell state of the model forward hidden cell in spatial attention, and each attention weight is obtained at moment *t*.$\;x_{t}^{\prime }$ is the result of the calculation of the spatial attention on water quality attributes, the spatial attention structure is shown in Figure 6.

### 2.4. Network Output Module

**Figure 7:**
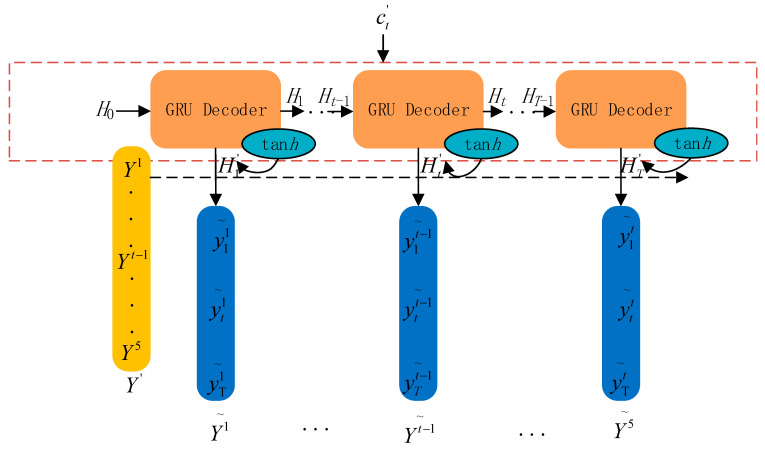
Output module.

**Figure 8:**
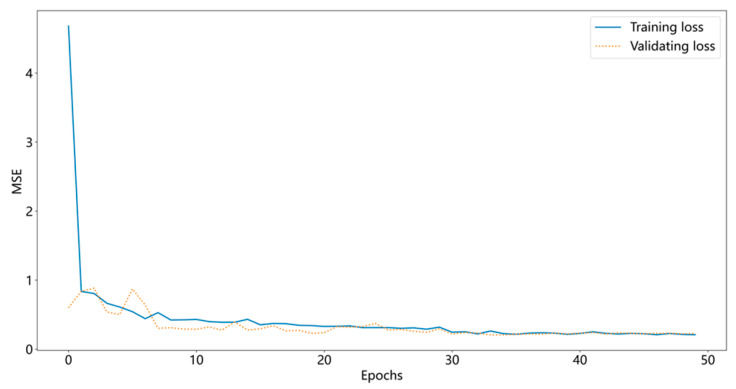
Mean square error of the prediction model.

The network output module mainly uses the water quality characteristic information learned by the attention mechanism to predict the key parameters of the water quality. The structure is shown in Figure 7. The output layer of the network is 5 unit nodes; the hidden state *H_t_*_−1_ of the previous moment and the predicted value $Y^{\prime }$ generate the next moment. The GRU decoder has hidden state *H_t_*; the combination of parameters *W_y_* and *b_y_* is [*H*_t_,$\;c_{t}^{\prime }$], and the weights $v_{y}^{T}$ and bias $b_{y}^{\prime }$ are used to generate the final predicted value $\tilde{Y}$ as in Equations (20)–(22). The mean square error (MSE) is used as the network loss function as shown in Equation (23). The GED network calculates the expected value of the square of the error between the predicted and true values at each moment through the MSE quantitative metric and then adjusts the gradient through the optimizer adam to adjust the weights of the network model parameters. When the loss of network training is reduced to the lowest value, the predicted values of $\tilde{Y}$ are obtained. The training loss curve is shown in Figure 8.

(20) $$y_{t}^{\prime }=w_{t}^{T}[y_{t},c_{t}^{\prime }]+b$$

(21) $$H_{t}=f(H_{t-1},Y^{\prime })$$

(22) $$\tilde{Y}=v_{y}^{T}(w_{y}[H_{t},c_{t}^{\prime }]+b_{y})+b_{y}^{\prime }$$

(23) $$\mathrm{MSE}=\frac{1}{n}\sum_{i=1}^{n}{\left(\hat{y_{i}}-y_{i}\right)}^{2}$$

## 3. Results and Analysis

### 3.1. Experimental Data Acquisition

**Figure 9:**
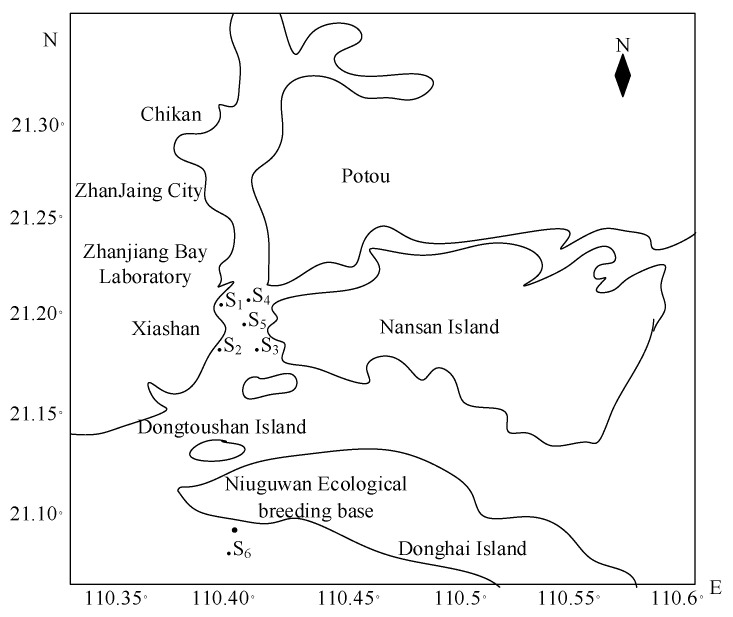
Monitoring stations near Zhanjiang Bay.

The seawater quality data were collected from six monitoring stations (S_1_, S_2_, S_3_, S_4_, S_5_, S_6_) in the sea near Zhanjiang Bay, Zhanjiang city. S_1_, S_2_, S_3_, and S_4_ were arranged around 25 × 25 m^2^ of an area of the sea, S_5_ was located in the middle of the area, in order to verify the generalization performance of the model, S_6_ was placed in the fixed area of the ecological mariculture institute in Niugu Bay, and each station meter was placed at a depth of 0.2 m from the water surface, as shown in Figure 9. The data of 45 water factors at each site were collected from 1 March 2017 to 15 November 2021, with a total of 12,516 sample sets collected, and the collection period was once per day, with sites S_1_, S_2_, S_3_, and S_4_ as the training set, with a total of 8344 sample sets, and sites S_5_ and S_6_ as the test set and validation set, with 2086 sample sets for each test set and validation set.

The experiments were conducted using an intel(R) Core(TM) i5-6200 CPU@2.30GHZ processer, with 8 GB of memory, running Windows 10 as the operating system, a programming language written in Python 3.6, and an integrated development environment of Pycharm Community 2020.2.1, with Keras in the program implementation.

### 3.2. Parameter Setting and Evaluation Index

**Figure 10:**
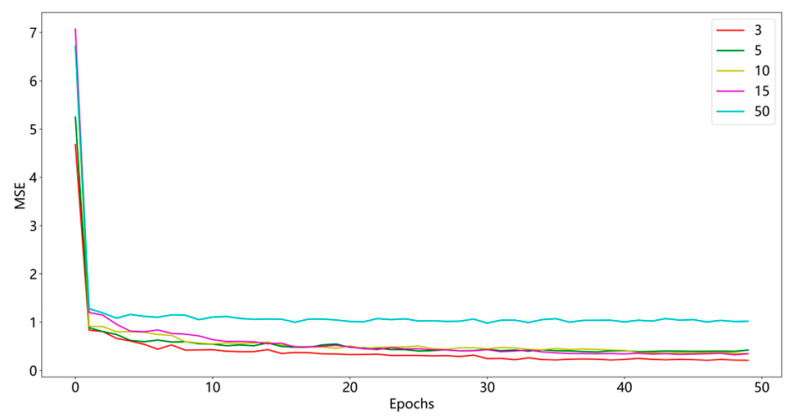
Mean square error of the model in different layers.

The learning rate (initialized to 0.01) is automatically adjusted according to the model training to make the gradient decrease by a factor of 100 for every 10 iterations. During the training process, to prevent the model from overfitting, the dropout layer is set to 0.5, which is located in front of the output layer. The batch size is set to 64, epochs are set to 50, the network optimizer is adam, and in the first layer, the number of hidden units in the GRU network is 100, as shown in Table 1, and the number of hidden units in the second and subsequent layers is 200. It is verified that the model sets the lowest MSE for the number of layers of GRU network layers to 3, as shown in Figure 10. The sample set is set at 80% as the training set and 20% as the test set, where the calibration sample set is 20% of the training set. By setting different network layers to compare the convergence performance of the prediction model, the EEMD algorithm noise reduction data process will set the original data to 95 of the amount of information retained.

In this paper, three quantitative evaluation indexes were selected to measure the predictive ability of the model for the key parameters of the seawater quality, such as the root mean square error (RMSE), mean absolute percentage error (MAPE), and coefficient of determination (*R*^2^), which are calculated as Equations (24)–(27).

(24) $$\mathrm{RMSE}=\sqrt{\frac{1}{n}\sum_{i=1}^{n}{\left(y_{i}-y_{i}\prime \right)}^{2}}$$

(25) $$MAPE=\frac{1}{n}\sum_{i=1}^{n}\frac{\left|y_{i}-y_{i}{}^{\prime }\right|}{y_{i}}$$

(26) $$R^{2}=1-\frac{\frac{1}{n}\sum_{i=1}^{n}{\left(y_{i}-y_{i}^{\prime }\right)}^{2}}{\frac{1}{n}\sum_{1}^{n}{\left(y_{i}-\bar{y}\right)}^{2}}$$

(27) $$\bar{y}=\frac{1}{n}\sum_{i=1}^{n}y_{i}$$

In the above equations, *n* is the number of samples in the model test set, $\bar{y}$ is the mean of the actual values, and $y_{i}^{\prime }$ and *y*_i_ are the predicted and true values, respectively. If the RMSE and MAPE are smaller and the *R*^2^ is larger, it means that the model has better comprehensive prediction performance.

### 3.3. Data Processing and Analysis

**Figure 11:**
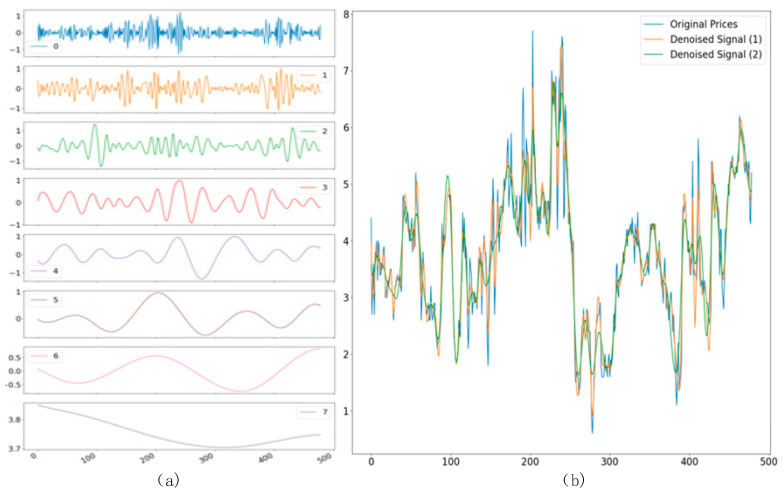
(**a**) Signal decomposition of water quality parameters (**b**) Water quality parameter signal denoising.

(1) Correlation analysis and dimensionality reduction. As the sample data reached 45 dimensions, if the water quality index data with little or no impact on the prediction results are used as model input, this will reduce the accuracy of the model prediction, so there is a need for the sample data to reduce the dimensionality. To avoid the PCA due to too much missing information, we used a combination of the missing value ratio and PCA. (1) Through the missing value analysis, we removed the missing value ratio of 10% of the variables, and then used the PCA algorithm to process water quality variables to obtain 10 variables. (2) We used IBM SPSS Statistics for principal component analysis to establish the principal component matrix. (3) To determine the eigenvalues of the correlation coefficient matrix and calculate its contribution rate, we extracted the cumulative contribution rate of 80% or more of the principal component variables as network input (the first five principal components’ cumulative contribution rate of 83.3% indicated that it can reflect most of the information of the original data). (4) To obtain the matrix of principal component coefficients, see Table 2, we calculated the coefficient > 0.8 of water quality parameters for prediction, including dissolved oxygen (DO), the chemical oxygen demand (COD), total phosphorus (TP), ammonia nitrogen (AN), and pH. (5) Through further correlation between the five key parameters such as dissolved oxygen in the analysis of water quality based on Pearson’s correlation analysis (PCA), it was concluded that dissolved oxygen was moderately correlated with pH and ammonia nitrogen, and the other factors were weakly correlated with each other.

(2) Ensemble empirical modal decomposition. Using the EEMD algorithm for each site to collect water quality data for the noise reduction process, with site S5 data as an example, the noise reduction steps are described in Section 2.1. In addition, data decomposition and the effect after noise reduction are shown in Figure 11a,b, respectively. Site S5 data were decomposed into six components and a residual, rounding off the components containing noise, reconstructing the signal IMF components, which were repeated two times to obtain the noise reduction water quality data signal (1) and (2) and the original signal curve for comparison. The curve fit was very good, which also showed that the original information characteristics were retained in the noise reduction process, excluding the water quality noise.

### 3.4. Ablation Experiments

To verify the effectiveness of the PCA–EEMD–CNN–Attention–GED prediction model proposed in this study, based on the water quality sample data from station S_5_ in the sea area near Zhanjiang Bay, this study uses the following four ablation experimental schemes. (1) Replacing the GED network with LSTM; (2) Removing the attention mechanism; (3) Removing both Attention and CNN; (4) Leaving the data unprocessed by PCA and EEMD algorithms (raw data).

**Figure 12:**
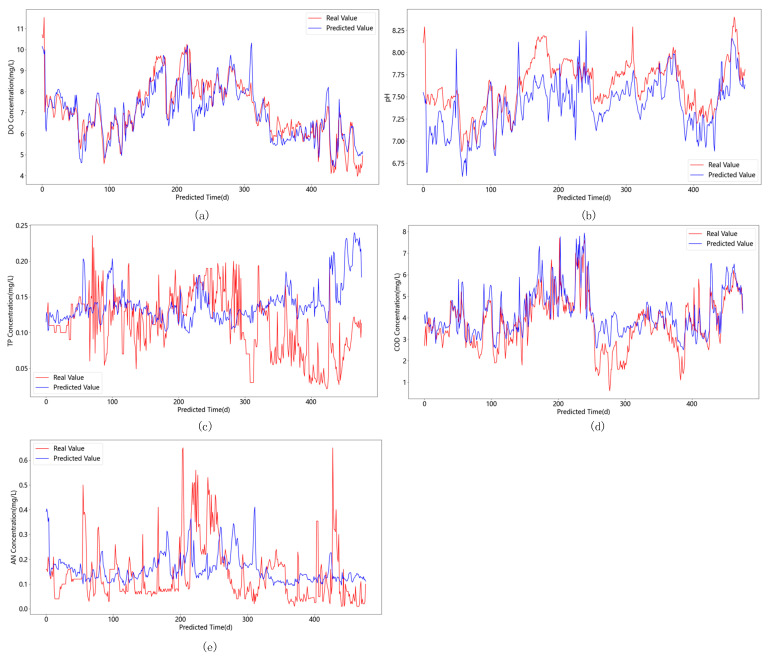
(**a**) Real and predicted values of Do concentration; (**b**) Real and predicted values of pH; (**c**) Real and predicted values of TP concentration; (**d**) Real and predicted values of COD concentration; (**e**) Real and predicted values of AN concentration.

The experimental results of the above four ablation experimental schemes are shown in Table 3. The PCA–EEMD–CNN–Attention–GED prediction model was compared and analyzed with the PCA–EEMD–CNN–Attention–LSTM prediction model and the CNN–Attention–GED prediction model, and the prediction accuracy was improved by 0.86% and 1.34%, respectively, when the prediction sequence was set to 1, and when the prediction sequence was set to 7, the prediction accuracy improved by 0.75% and 0.55%, and when the prediction sequence was set to 15, the prediction accuracy improved by 0.72% and 1.92%, respectively. Then, the prediction accuracy of this paper improved by 0.73%, 0.7%, and 1.68% when the prediction sequence was set to 1, 7, and 15, respectively, when the prediction model was compared with the PCA–EEMD–CNN–GED prediction model. Finally, the prediction model in this paper was compared with the PCA–EEMD–GED prediction model again, and its prediction accuracy improved by 0.89%, 1.03%, and 2.11% when the prediction sequences were set to 1, 7, and 15, respectively. Comprehensive experimental analysis showed that the comprehensive performance of the prediction model proposed in this paper was better than the PCA–EEMD–CNN–Attention–LSTM prediction model, CNN–Attention–GED prediction model, PCA–EEMD–CNN–GED prediction model, and PCA–EEMD–GED prediction model, and the predicted values of the model for key parameters of water quality better fit with the real values, as shown in Figure 12a–e. Further analysis of water quality key parameters was performed. From the comparison of the predicted and actual values of the five key factors of water quality, the actual and predicted values of the total phosphorus content in Figure 12c during the 300–500 scale curve fit the error; the deviation between the predicted and actual values of other factors was very small, the prediction effect was better, and the prediction accuracy and reliability were in line with the requirements of water quality prediction.

### 3.5. Comparison Experiments

In order to verify the robustness of the model proposed in this study and compare it with the proposed model, the S6 data sample set of the Niuguan Bay ecological aquaculture water quality site was used as the input data of the model, and the ECA–Adam–RBFNN, PSO–SELM–PLS, and wavelet transformation-depth BI-S–SRU prediction models were selected for comparative analysis. The prediction sequence was set to 20, and other parameters were set as the same as those of the model proposed in this study; the quantitative results were analyzed as shown in Table 4. The experimental results showed that the PCA–EEMD–CNN–Attention–GED prediction model was lower by 29.5%, 15.1%, and 48.5%, respectively, in terms of RMSE compared with the ECA–Adam–RBFNN, PSO–SELM–PLS, and wavelet transform-depth Bi–S–SRU prediction models; lower by 14.6%, 2.8%, and 15.4% in terms of MAPE, and lower by 1.43%, 0.44%, and 2.96% in R^2^. In summary, this paper verified the feasibility of the PCA–EEMD–CNN–Attention–GED prediction model, showing that the model has high prediction accuracy and robustness and can provide a decisive basis for mariculture water quality warning and water quality regulation management.

## 4. Discussion

The work done in this study proposing the application of a combined model for the prediction of seawater quality parameters is meaningful, but there are several more aspects to explore.

(1) Discussion from the perspective of water quality parameters. In this paper, as the water quality parameters were obtained based on the needs of local farmers, only some physical and chemical factors were considered. Based on this study, the future of biological factors and other chemical and physical factors to be considered, such as eight heavy metals (mercury, cadmium, lead, chromium, copper, zinc, nickel, arsenic), sedimentation, runoff, erosion, dissolved oxygen, decaying organic matter, pesticides, precipitation, climate, soil type, vegetation, geology, water flow conditions, groundwater and other factors.

(2) Discussion from the perspective of the research area. This study is only for seawater domain water quality parameters; the proposed combined model can give reference to freshwater domain water quality prediction, and further extension to freshwater domain water quality prediction needs to be verified.

(3) Discussion from the perspective of model optimization. The PCA–EEMD–CNN–Attention–GED prediction model proposed in this paper has the ability to extract water quality spatio–temporal sequence features and further use the literature [26] multi-layer approach to optimize this paper’s model to extract water quality three-dimensional spatial features.

## 5. Conclusions

This paper proposes a PCA–EEMD–CNN–Attention–GED mariculture water quality prediction model, which improves the prediction accuracy of the traditional water quality model, has the advantages of extracting water quality spatio–temporal sequence characteristics, and high accuracy, and can be widely used in the field of seawater quality monitoring to meet the needs of farmers for farming water quality monitoring and changing the traditional way of on-site water quality monitoring. Due to the actual needs of local farmers for water quality parameters, only existing data were used to construct the model, and some chemical, physical, and biological factors were not taken into account. The next step is to first collect data on other water quality parameters, then verify the performance of this paper’s model in freshwater water quality prediction, and finally optimize this paper’s model to build a prediction model that can extract three-dimensional spatial characteristics of freshwater water quality and explore the changes in freshwater water quality parameters in three-dimensional space.

## Figures and Tables

**Table 1:** Comparison of different hidden unit models.

Models	Model of Hidden Units	RMSE	MAPE	R^2^
PCA–EEMD–CNN–Attention–GED (GRU Encoder-Decoder)	16	0.821	0.564	92.67
	64	0.778	0.549	93.36
	100	0.644	0.516	94.25
	200	0.694	0.540	93.92

**Table 2:** Principal component coefficient matrix.

Indicators	Component 1	Component 2	Component 3	Component 4	Component 5
pH	−0.395	0.813	0.217	−0.011	0.027
Turbidity	−0.636	0.172	−0.255	0.222	0.247
Dissolved oxygen	0.443	0.461	0.882	0.011	−0.076
Water temperature	−0.768	−0.325	0.471	0.148	0.078
Electrical conductivity	0.571	0.219	0.092	0.687	0.232
Chemical oxygen demand	0.809	−0.094	0.253	0.184	−0.197
Ammonia nitrogen	−0.358	−0.079	0.873	−0.169	0.515
Total phosphorus	0.067	0.893	0.522	−0.294	0.149
Potassium permanganate	0.423	−0.347	−0.319	−0.421	0.586
Redox potential	−0.256	0.210	−0.734	−0.164	−0.415
Eigenvalue	2.934	2.429	2.194	0.894	0.748
Contribution rate/%	26.7	48.8	67.5	74.7	83.3

**Table 3:** Algorithm comparison.

Models	In-Out	RMSE	MAPE	R^2^ (%)
PCA–EEMD–CNN–Attention–GED	1-1	0.246	0.307	97.80
	7-7	0.692	0.560	94.23
	15-15	0.832	0.596	93.57
PCA–EEMD–CNN–Attention–LSTM	1-1	0.347	0.347	96.94
	7-7	0.834	0.605	93.48
	15-15	0.896	0.623	92.85
PCA–EEMD–CNN–GED	1-1	0.338	0.353	97.07
	7-7	0.843	0.622	93.53
	15-15	1.021	0.642	91.89
PCA–EEMD–GED	1-1	0.345	0.362	96.91
	7-7	0.757	0.548	93.20
	15-15	1.213	0.744	91.46
CNN–Attention–GED	1-1	0.394	0.418	96.46
	7-7	0.747	0.563	93.68
	15-15	0.975	0.637	91.65

**Table 4:** The prediction sequence for a 20–20 model comparison.

Models	In–Out	RMSE	MAPE	R^2^ (%)
PCA–EEMD–CNN–Attention–GED	20–20	0.878	0.594	92.23
ECA–Adam–RBFNN [11]	20–20	1.173	0.740	90.80
PSO–SELM–PLS [12]	20–20	1.029	0.622	91.79
Wavelet Transform-Depth Bi–S–SRU [14]	20–20	1.363	0.748	89.27

## Data Availability

The data presented in this study are available on request from the corresponding author. The data are not publicly available due to privacy restrictions.
